# New Biomarkers of Innate and Adaptive Immunity in Infectious Diseases

**DOI:** 10.1155/2017/7047405

**Published:** 2017-12-14

**Authors:** Sergey Morzunov, Varough Deyde, Levon Abrahamyan

**Affiliations:** ^1^University of Nevada, Reno, USA; ^2^CDC, Pretoria, South Africa; ^3^Université de Montréal, Montreal, Canada

Despite dramatic achievements in the field of infectious disease control and prevention in economically developed countries, infectious diseases still remain the leading cause of morbidity, disability, and mortality worldwide. According to the World Health Organization (WHO), 15 million fatalities were attributed to infectious diseases in 2010. It is extremely alarming that the WHO predicts 13 million infectious disease-related deaths for 2050 [1]. In particular, emerging infectious diseases are on the rise, which can be explained by many factors like microbial adaptation to new hosts, selective pressures of the treatment, migration of the natural hosts, and so on. Emerging infectious diseases often have deadly consequences which may threaten the existence of humanity. Although much has been learnt about the pathogenesis of infectious diseases, reliable early diagnosis and effective treatment for many of these diseases are still not available. These limitations could be explained by our limited knowledge of the molecular host-pathogen interactions and immune response to many particular infections, which hampers the discovery of the early diagnostic biomarkers.

Recent advances in molecular biology and immunology resulted in a rapid expansion in the field of immune biomarkers. These biomarkers can help make an early diagnosis, evaluate the efficacy of treatment, and improve or predict the disease outcome. The increase of emerging infections and spread of the antibiotic-resistant bacterial strains make the search for biomarkers especially urgent. Recently, the U.S. Food and Drug Administration addressed the need for Immune Biomarkers regarding development and approval of the new diagnostics as essential for improving the treatment of infectious diseases. The ongoing search for biomarkers is wide and includes correlation analysis between clinical presentation and genetic mutations, cytokines, receptors, growth factors, and so on found in the host or the infectious agent. Despite extensive research, the need for novel biomarkers remains critical. For example, not much is known about immune biomarkers for such emerging/reemerging infections as Zika virus-caused disease and Ebola hemorrhagic fever. There is also ongoing search for biomarkers of the immune dysfunction in HIV and severe dengue disease. Novel biomarkers for cytomegalovirus and *Mycobacterium tuberculosis* provide useful information on the human host response to the corresponding infections.

We invited prospective authors to contribute original manuscripts, case reports, clinical trials, and reviews focusing on genetic aberrations, cytokines, growth factors, and other small biologically active molecules as potential biomarkers in infectious disease. As a result, the manuscripts selected for this special issue reflect the current diversity of this research field.

The investigation of C. C. Emene et al. presents genetic markers associated with an increased chance of developing erysipelas. The authors also identified genetic markers associated with the localization of the infection on different parts of the body. In addition, elevated serum cytokines IL-1*β*, CCL11, IL-2R*α*, CXCL9, TRAIL, PDGF-BB, and CCL4 were found in erysipelas patients. Furthermore, increased serum levels of IL-6, IL-9, IL-10, IL-13, IL-15, IL-17, G-CSF, and VEGF were detected in patients with recurrent erysipelas. Finally, correlation analysis revealed that level variations of IL-1*β*, IL-7, IL-8, IL-17, CCL5, and HGF were associated with SNPs in the SOD2 gene, while variations of PDFG-BB and CCL2 were found to be associated with SNPs in the CAT gene.

N. Molaee et al. investigated the immune response in mice to the *Vibrio cholera* recombinant pili and flagella proteins. High levels of IL5 and low levels of IFN*γ* observed in response to injection of FlaA and TcpA suggest that these proteins stimulate immune responses toward Th2, while high levels of IL5 and high IgG1 antibody titer observed after injection of TcpB suggest that it mostly directs immune responses toward Th1. The data provided on the immune response in humans is identical; combining these recombinant antigens with potential *Vibrio cholera* vaccine can cause higher immunogenicity and better protection against cholera.

Y. A. Tyurin et al. evaluated a cytokine profile in nasal secretion and blood serum of the patients with seasonal (SAR) and perennial (PAR) allergic rhinitis. The authors showed that increased GM-CSF production is maintained in the patients with PAR sensitized to the house mite allergen components. A higher production profile of TNF*α* and TSLP was also detected in nasal secretion in the patients with PAR and additional high sensitization to SEs. Sensitization to mold fungi allergen components was significantly higher in patients with SAR. In particular, these patients displayed a high level of sensitization to the *Aspergillus fumigatus* component m3. A negative correlation was demonstrated between serum TGF-*β* level and the age of patients with SAR. TGF-*β* can inhibit the immune response and proliferation of immunocompetent cells. Thus, along with other clinical trials, the study performed and clarified some aspects of the molecular pathogenesis of human allergic rhinitis.

In the second investigation of Y. A. Tyurin et al., the correlation between polymorphisms of the Toll-like receptor genes TLR2 and TLR4 in relation to clinical and immunological parameters in atopic dermatitis patients was analyzed. Observed dysregulation of cytokine production (IL-4, IL-10) in the patients with heterozygous polymorphic genotypes probably reflects an imbalance of Th1/Th2/Th17 regulation of immune response in these individuals.

N. S. Zakharchenko et al. evaluated the antimicrobial efficacy of the *Kalanchoe pinnata* extract containing cecropin P1 (CecP1). *K. pinnata* extracts were tested for the treatment of wounds infected with *Candida albicans*. The therapeutic efficacy of *K. pinnata* extract was comparable with that of a commercial fungicide clotrimazole. However, *clotrimazole* neither facilitated wound healing nor caused remodeling of the scar matrix. The improved therapeutic effect of the *K. pinnata* extract was attributed to a synergism between the fungicide activity of CecP1 and wound healing (antiscar), revascularizing, and immunomodulating effect of natural biologically active components of *K. pinnata*. Taken together, data presented suggest that CecP1-enriched *K. pinnata* extracts could be a candidate drug for the treatment of dermatomycoses and fungus-contaminated wounds.

In a related investigation performed by A. A. Lebedeva et al., the therapeutic effect of *K. pinnata* water extracts containing CecP1 on healing of wounds contaminated with *S. aureus* or combined *S. aureus* with *P. aeruginosa* was evaluated. The efficacy of *K. pinnata* extract treatment of *S. aureus* turned out to be equal to that of cefazolinum; however, it performed better than cefazolinum when treating the wounds simultaneously contaminated with *S. aureus* with *P. aeruginosa*. *K. pinnata* extracts (both wild-type and transgenic) did not exhibit general toxicity while accelerating wound recovery. Immunomodulating and microbicide activity of *K. pinnata* synergize with microbicide activity of CecP1 accelerating elimination of bacteria.

In the study of E. V. Martynova et al., several important correlations were detected for the nephropathia epidemica (NE) patients. In particular, elevated levels of triglycerides and decreased HDCL were found in NE patients, while total cholesterol did not differ between NE cases and controls. Higher triglyceride levels were found in males as compared to female NE cases. Furthermore, data indicated that high triglycerides are associated with the lowest thrombocyte counts and high serum VEGF, as well as a high severity score. On the other hand, low levels of triglycerides were associated with upregulated IFN-*γ* and IL-12. Activation of the Th1 type immune response was suggested, since IFN-*γ* and IL-12 levels were increased in patients with lower severity scores. This relationship strongly indicates that Th1 lymphocytes play a protective role in NE. These data advance our understanding of NE pathogenesis and establish a clear link of the high triglyceride level with the severity of the disease.

Kh. S. Khaertynov et al. evaluated the serum levels of the proinflammatory cytokines (TNF-*α*, IL1-*β*, and IL-6) and the anti-inflammatory cytokines (IL-4 and IL-10) in neonatal sepsis cases. It was demonstrated that both the late onset of sepsis (LOS) cases and the early onset of sepsis (EOS) cases were characterized by increased serum level of TNF-*α*. However, increased serum levels of IL-6 and IL-10 were found in LOS cases only. Moreover, levels of the proinflammatory cytokines, such as TNF-*α* and IL-6, were elevated in the acute phase of sepsis, whereas the anti-inflammatory cytokines, such as IL-10, were substantially upregulated during the postacute phase of the disease. The authors concluded that the analysis of serum cytokines can provide valuable information when determining the most effective therapy for treating neonatal sepsis.

In the study by D. Akberova et al., serum cytokine levels were investigated in several autoimmune diseases including autoimmune liver diseases (AILD), autoimmune hepatitis (AIH), and overlap syndrome. AILD cases were characterized by increased levels of IL-6, IL-8, and TNF-*α* as compared to controls. Statistical analysis revealed a correlation between high IL-8 and diagnosis of AILD, AIH, and overlap syndrome.

Due to an excellent response, this special issue includes a number of the original research articles aimed at the detection and analysis of the various potential biomarkers (genetics, proteomics, cytokines, growth factors, hormones, and so forth) of infectious disease contributing to diagnosis, treatment, and prophylaxis. The analysis of these novel biomarkers was based on the bioinformatics approaches that were employed to characterize biomarkers associated with the clinical presentation, severity, and treatment efficacy of infectious diseases. In addition, this issue includes data on novel therapeutic approaches for the treatment of infectious disease using both novel and presently known biomarkers. Finally, new data on the biomarkers of the immune evasion and development of resistance to viral and microbial pathogens are presented in this issue.

*Sergey Morzunov* *Varough Deyde* *Levon Abrahamyan*
